# Hospital Gangrene in the U. S. Military Hospital, Memphis, Tenn.

**Published:** 1864-01

**Authors:** C. H. Cleaveland

**Affiliations:** A. A. S., U. S. A.


### CHICAGO MEDICAL JOURNAL.

VOL. XXI.] JANUARY, 1864. [No. 1.

### ORIGINAL COMMUNICATIONS.

The following report was prepared by Surgeon Cleaveland at the request of Dr. Brinton, as a continuation to the "Surgical History of the War." As it may be of great service, the author has, at our request, furnished us with a copy for present publication.—[*Eds. Journal.*]

### HOSPITAL GANGRENE IN THE U. S. MILITARY HOSPITAL, MEMPHIS, TENN.,

By C. H. CLEAVELAND, A. A. S., U. S. A.

*General History.*—Although I have been at no inconsiderable pains to obtain a history of Hospital Gangrene, as it has appeared in the Military Hospitals of this city, I am convinced that I have been unable to collect all the facts needed for a complete history, and therefore shall confine myself mainly to such as fell under my own personal observation.

The first cases of this disease which fell under my notice were those of Lieuts. Alban, Deelomyer, Letnir, and Davis, who were brought from Vicksburg to Officers' Hospital, of which I then had charge. They were received from the Hospital boat on the evening of the 27th of May, and each had gangrene of several days' standing when admitted, and were expected to survive but a very few hours. In fact Lieut. Letner, who had gangrene of the stump of the left leg, died on the 29th, the second day after admission, and Lieut. W. H. Alban on the 1st of June, the fourth day after admission.

Although the flaps sloughed away from the stump of Lieut. Deelomyer so as to leave the bone uncovered for at least two inches, still the periosteum remained vitalized, the gangrene was quickly arrested by the application of Labarraque's Chlorinated Sodæ Solution, under the vigilant care of Cadet Randall, who attended to the dressing of this patient. Granulations sprang up all over the surface of the bone, a fine conical stump was formed, and on the 12th of June, about a fortnight after admission, he was dismissed on leave of absence and went to his home.

About this time, and later, quite a number of cases occurred in the different hospitals, but their nature was not always understood by attendant surgeons. When attention was specially called to this disease by Surgeon Irwin, Superintendent of Military Hospitals, cases were found in the Union Hospital to the number of twelve—June 25th—and soon thereafter at the Jackson, Gayoso and Overton Hospitals. The Surgeon in charge of Adams Hospital reports the case of a man who came from the vicinity of Vicksburg on the 18th of February. He says that is the only case which has occurred in that Hospital this year, a statement evidently erroneous, as Corporal Fuller was sent here from there with gangrene of right ankle on the 9th of August. A similar error occurred in the report of the Surgeon in charge of the Webster Hospital, who says but one case has occurred in his Hospital, and yet he has sent to us Privates Alt, McEvoy, Cloud, McClure, Butler and Overt, all with unmistakable gangrene. From the Overton Hospital we have received fifteen (15) patients, and how many more have had the disease in that Hospital I am unable to determine, as I get no response to my applications for information. From the Jackson Hospital we have received eleven

(11) patients, but from that Hospital, also, have been unable to obtain any farther information.

Up to the 1st of September forty-six (46) patients have been sent to us from other Military Hospitals, having gangrene, which I have reasons for supposing were not more than half the cases that have occurred in the Hospitals. I know of seven (7) cases occurring at the Officers' Hospital which were retained there for treatment as we had no special accommodations for officers.

Not only have there been manifest errors in the reports from different Hospitals in regard to the number of patients who have had gangrene, but the date of its appearance has been incorrectly reported. Early in the month of June the wards of Union Hospital were filled with patients from down the river, and immediately Cadet Randall reported to me that gangrene was among them. Surgeon Irwin states that according to information in his possession, gangrene appeared *first* in the Union, then in the Jackson, the Gayoso, and Overton Hospitals, and his order for organizing and opening Church Hospital was issued on the 19th of June, one week before the disease was *reported* to have appeared in Union Hospital.

Although the order for opening and establishing this Hospital was issued on the 19th of June, yet owing to delay in the Quartermaster's department no patient was received until the 30th of July, and the two days of July are included in the report for the month of August. During its organization, and until the 7th of August, I was in charge of this Hospital, when Surgeon George R. Weeks was assigned to its temporary charge for the special purpose of testing the use of Bromine in the treatment of gangrene.

Until Surgeon Weeks came, we had no Bromine. One ounce had been furnished to the Hospital, but after the dressers had used it once it unaccountably disappeared. During that time reliance was placed mainly in the local use of Labarraque's Solution of Chlorinated Soda applied in its full strength, although the use of Nitric Acid, Acetic Acid, Sulphate of Zinc, and Muriated Tincture of Iron was resorted to in some cases.

The favorable result which followed the use of this preparation in the case of Lieut. Deelomyer, at Officers' Hospital, gave me considerable confidence in it, both in gangrene and in erysipelas, which confidence is shown by the Hospital records not to have been misplaced, as every case of erysipelas sent to us was cured by it alone, in from three to five days. Private Samuel Arbothnot was sent here from Jackson Hospital with gangrene in the palm of the left hand, July 30th, and was returned cured of the gangrene, August 8th. Robert Graham was admitted from the Union Hospital on the 31st of July with gangrene of the right arm, and returned with no vestige of gangrene remaining, on the 4th of August. Private William Goff, admitted from Union Hospital, Aug. 1st, with gangrene of right leg, was returned cured, Aug. 4th. Private D. L. Twiman was sent here from the Overton on the 31st of July, with gangrene of the right thigh, and returned cured of gangrene on the 5th of August. Private Fred Louis came from the Jackson Hospital with gangrene of the right hand on the 30th of July, and was returned cured, on the 4th of August. Other similar cures have followed the persistent use of the Chlorinated Soda Solution since that date.

On the 8th of August, when Surgeon Weeks took charge of the treatment he brought with him, from Surgeon Irwin, five (5) ounces of pure Bromine, which supply was exhausted on the 16th of August, and no more has been had since. On the 25th of August, Dr. Warriner, agent of the U. S. Sanitary Commission, gave to the Hospital five bottles of Prof. J. Lawrence Smith's Compound Solution of Bromine, which is now used in place of the pure article, but not to the satisfaction of any one connected with the Hospital, as it evidently is by no means equal in efficiency to the Bromine in full strength.

During the week that pure Bromine was applied it was found, as the Hospital records show, that usually, one single application sufficed to completely eradicate the gangrene. In cases, of apparent failure, it was afterwards found that there were some parts deep, between muscles, under the integuments, or in sulci or sinuses, which the Bromine did not reach. While we had no Bromine, and also, when we had only the Compound Solution, we found it much more difficult, and in some cases quite impossible, to eradicate the disease, mainly from the impossiblity of getting the remedies directly in contact with the entire surface affected.

Up to the 1st of September, forty-six (46) cases of genuine hospital gangrene have been treated in this Hospital. Of that number thirteen (13) died, but none directly from the gangrene. During the month fifteen (15) have been cured, and returned to the Hospitals from which they came, and eighteen (18) still remain under treatment, most of whom are entirely free from gangrone, but are not sufficiently restored in health to leave the Hospital. Some of these will die from the effects of gangrene on the system.

*Diagnosis.*—Hospital Gangrene as it has been presented to us at this Hospital, has assumed a great variety of appearances. In the earlier stages it has appeared as a dusky, almost black, mass of dead and rotten flesh, occupying the seat of the disease, surrounded by a reddish ring of slightly swollen integuments, which ring varies from one to five lines in breadth, while the adjunct tissues do not appear to be affected by the disease, except that they, as well as the whole body, contain less heat than is natural in healthy structures. Often the disease has burrowed under the integuments bordering a wound, and when the skin dies it loses its epidermis, the true skin becomes reddish, greenish, purplish, or black. When a surface already divested of its skin is affected with the disease (and most of the cases at this Hospital are truly traumatic), the first invasion appears by giving to the surface an ashy gray color, with a pultaceous consistency, and the *peculiar* odor of spoiled meat, by which the disease is readily recognized. This pultaceous, ashy appearance of the surface of wounds presents itself when the areolar tissue is attacked.

When the muscular tissue becomes affected, and when small blood vessels have become ruptured, a dark, grumous, almost black, dirty appearance of the diseased surface is presented, accompanied by a powerful fœtid odor, and usually with an invasion of the disease under the skin, and between the muscles and tendons in the connective tissue, much beyond the boundary of the disease as it appears on a superficial examination. In the case of Private Milseps, who was wounded at Port Hudson on the 27th of May, with a minie ball in the right thigh, and the ball was allowed to remain in until he came here, the disease burrowed in the fascia of the thigh, until his whole thigh appeared gangrenous in the deep-seated parts, and he died suddenly from arrest of circulation. Post mortem examination revealed a detached thrombus in the left auricle of the heart, plugging up the artery, which weighed four hundred and eighty-two (482) grains. One patient, Private T. D. Beggs, who was wounded high up in the right calf by a musket ball, at Helena, on the 4th of July, and brought here on the 31st, had the disease following down the tendon to the os calcis, which was necrosed. Although the entire extent of the leg was laid open, it was too late to save the patient who has since died (Sept. 2d) from the absorption of gangrenous matter. I removed from his thoracic duct a thrombus near six inches in length. These two cases are but extreme illustrations of the tendency of the disease to extend itself in the areolar tissue along plains, and between more solid organs and structures.

But muscles, vessels, nerves, tendons, periosteum and bone are often invaded, when the part affected turns dark, emits a peculiar (gangrenous) odor, dies, softens, and finally liquifies when not removed in the form of slough, or by the dressers's instruments.

*Preparation of Wound.*—I am fully satisfied that many surgeons have failed in the treatment of Hospital Gangrene, because they have not fully perceived the necessity of thoroughly cleansing the wound of all dead and diseased matter previous to the use of all local applications. Private James McEvoy, who was wounded at Raymond, Miss., on the 12th of May, and had his right arm amputated at about its middle, had gangrene of the stump about the 20th of June, and was sent here the 10th of August, with a statement that Bromine had been carefully applied daily for eight days. On examination it was found that there were at least two inches in extent of dead structure on the end of the stump, and the Bromine had been applied to that and not to the live tissue. All the dead and dying structures were carefully dissected from the surface of the stump, removing a large quantity of putrescent matter, and then Bromine in full strength was cautiously applied to the entire surface, and the disease was at once arrested. He remained ten days, and then was returned to Webster Hospital.

When patients are first brought here it is often necessary to place them under the influence of chloroform, while their wounds are being prepared and to obtund the pain caused by the remedies applied, and afterwards chloroform is not refused them if the dressing is likely to be very painful. The wound is washed by means of a stream of warm water projected from a syringe; all the pultaceous and grumous matter removed with soft cloths; the more tenacious diseased portions taken away with the forceps, the scissors, and scalpel; and finally all blood, water, moisture, and semi-fluid matters under the skin, or between muscles or tendons, are removed by a little swab, made by winding a short strip of soft cloth around a little stick. If the disease has burrowed under the skin it is better to at once open up all such retreats as well as to carefully pare off the diseased portions of the skin on the edges of the wound, that the agent applied may come directly in contact with the vitalized structures in the parts affected. If this thorough preparation of the wound is not attended to, through carelessness or oversight, the progress of gangrene is not likely to be stayed, whatever may be the remedy applied.

Soap-suds as a wash does not answer for gangrenous ulcers as well as pure warm water, for it appears to combine with the fluids of the surface producing a slimy mixture which it is difficult to remove. A diluted solution of Labarraque's Chlorinated Soda has been found to answer admirably in these cases, for when applied in a gentle stream from a syringe, it can be made to penetrate into all the recesses of the disease, destroying all odor, and dissolving and removing some of the dead matter attached to the surface of the ulcer. At about the strength of sea water it seems to properly stimulate the structures as well as to cleanse them. An ordinary hard-rubber syringe with the little swabs already described, have proved preferable to sponges or cloths for cleansing tender and delicate surfaces.

*Local Applications.*—Any substance which has the chemical power of decomposing the specific virus of gangrene, so that it no longer exists as gangrene, must possess the property of a curative agent in this disease, provided it can be brought in sufficient quantities in direct contact with each atom of gangrene matter, and, provided also, that it does not possess sufficient power to overcome and destroy the vitality of such still vitalized structures and tissues as we wish to preserve. We have made trials of Nitric Acid, until we have become satisfied of three things in regard to it. It will destroy all gangrenous matter with which it is brought in contact, in its full strength. It is extremely difficult to apply it in its full strength to all parts of the disease in many cases, as in sinuses and sulci, when the moisture already in those parts may so dilute it as to render it inefficient. It is unsafe, for, if brought in contact with the live tissues, nerves, vessels, periosteum, or bone, it is almost certain to cause destruction to the parts and produce irreparable injury. Sulphate of Zinc in solution does not appear powerful enough to arrest the disease in many instances. Acetic Acid has not been applied in a sufficient number of cases to have led to any positive conclusion. The same may be said of the solution of the Chloride of Zinc. The Muriated Tincture as well as the solution of the Per-Sulphate of Iron have both been used, but mainly on bleeding surfaces and to check hæmorrhage from ruptured vessels, and while they may have a curative power, such property has not been so manifest as to give us much confidence in them as arrestors of gangrene.

Labarraque's Chlorinated Soda Solution has proved quite efficient, applied in its full strength, as an arrestor of gangrene. In all cases where the liquid could be thoroughly applied to the whole of the affected parts, one, or at most, three or four applications, appeared to eradicate the disease. But it could not always be applied to deep-seated parts, or within sulci, without its becoming diluted with the fluids already there,and such a quantity of the solution seemed to be demanded that it was liable to flow beyond the ulcer, and the healthy skin be blistered by it. But during nearly one-half the time this Hospital has been opened, we have placed our main reliance on this agent, and our confidence in it has not been shaken. In all cases of ulceration where no gangrene remains, we wash the surface once or twice a day with this solution diluted and saturate the dressings with it as often as they get dry, or once in two hours, and are well pleased with the result.

Bromine in its full strength, however, is the agent that has given us the most satisfaction. This does not appear caustic enough to destroy healthy tissues, but is of sufficient strength to unite with, and change all the parts affected with the disease. Of course a few drops will not unite with, and change a large amount of gangrenous material, but in all cases under my observation, when the dead matter had been properly dissected away, and the wound cleansed of its pultaceous and other dead matter with the syringe, the forceps, and the swab, and the surface properly dried with soft cloths, one application, or at most but two or three, have sufficed to eradicate all traces of gangrene, and leave the wound a simple ulcer. As has been observed, the wound should be properly and carefully prepared before the Bromine is applied, even if all the other necessary conditions are complied with, should there be moisture allowed to remain in the deeper portions of the sore, that moisture may so dilute the Bromine as to render it inefficient, or it may protect the subjacent parts from the remedy, and a point of gangrene will remain to infect the whole sore.

Bromine can be conveniently applied by means of little swabs, like those used in cleansing the wound. First, there should be prepared and at hand, a cloth spread with simple cerate, large enough to more than cover the affected surface; the ulcer should be cleansed and dried, and then, an attendant standing near the patient, holds the phial of Bromine from which he removes the stopper just long enough to allow the surgeon to insert into it a little swab, saturating it with the Bromine, which is at once applied to the ulcer. This operation is to be repeated until all parts of the sore are touched with plenty of Bromine, and to parts beyond the reach of the swab Bromine should be injected by means of a glass p.p. syringe, when the sore should be immediately covered with the cerated cloth, to prevent further evaporation of the Bromine, and to protect the ulcer from the atmosphere. Then the part may be dressed with cloths and bandages the same as for simple sores.

The pain which attends and follows the application of Bromine is quite severe, but not more severe nor of longer continuance than follows the application of milder agents. The sense of pain appears even somewhat obtunded by the action of the agent, and after a short time patients express themselves as being much easier than they have been for days.

To surgeons and attendants, Bromine, when incautiously used, proves very disagreeable, and that fact, doubtless, has prevented its use in many cases. If the vapor of Bromine is allowed to come in contact with the eyes and air-passages, such parts suffer severely from the irritation for several minutes, but soon all unpleasant effects pass away. But with proper precaution those parts need not be exposed to Bromine vapor, and ten or twenty patients may have Bromine applied to them in a morning without much discomfort to the surgeon.

Whatever agent is applied to a gangrenous surface, it does not appear desirable to disturb the dressing more than once a day. When Bromine is used it is usually necessary to wait two or three days for the surface of the sore to become thoroughly cleansed before it is again resorted to, but if there remains a decidedly gangrenous odor on the second day, or if it appears at any future time, the parts should be carefully examined and Bromine applied to all affected surfaces. Gangrene can be detected by the trained sense of smell more readily than in any other way, and for several days after the disease has disappeared wounds should be examined in this manner as well as by the eye, and all appearances of taint followed to their sources, and the disease eradicated.

On the second day, usually, after Bromine has been applied the surface of the ulcer looks dark and almost charred; on the third day the dead portions begin to liquify, and the fourth day the surface appears studded with healthy granulations.

After the gangrene is subdued, the wounds are dressed as simple ulcers, but in most cases the dilute Chlorinated Soda Solution is constantly applied, both as a slight stimulant to the newly-formed tissues, and to protect the wound from the virus which may be in the atmosphere of the Hospital.

I have made considerable use of Oakum, wet with a solution of Chlorinated Soda, or of the Chloride of Lime, to gangrenous ulcers *the day after* applying Bromine, and to the simple ulcer after the wound becomes free from gangrene, and am satisfied that in general it should be preferred to lint. It is easy of application, readily absorbs the excretions, and is easily removed when the wound again requires dressing. A very convenient way for the nurses to apply moisture to the sore, I have found to be by means of an ordinary hard-rubber syringe. The solution may be drawn into the syringe, the pipe of which can readily be inserted under the bandage, and the liquor applied directly to the Oakum, filling and covering the ulcer and moistening the entire dressing.

The Compound Solution of Bromine has been extensively used by us, and especially since our stock of pure Bromine became exhausted, but we do not consider it a good substitute for pure Bromine. It does not appear to be active enough to readily destroy the gangrene. It has been applied carefully, thoroughly and faithfully, day after day, by the swab and the syringe, into parts where the gangrene has burrowed under fascia, and along the tendons, as it is extremely apt to do, and directly to the walls of such extensions, when they have been laid open, by means of pledgets of lint, saturated with it, as well as in the same manner to the original ulcer, and yet we have sometimes failed for days to arrest the disease. I have made careful trial of both the Compound Solution of Bromine and Labarraque's solution of Chlorinated Soda, and can not percieve that the Bromine preparation possesses any valuable properties which the Chlorinated Soda has not. Both destroy the disease when applied to it, but the Compound Solution of Bromine causes the most pain, does not appear to be any more active than the Chlorinated Soda solution in full strength, and very readily loses its Bromine by vaporization, if not quickly and cautiously covered with a cerated cloth.

Pure, unadulterated Bromine seems endowed with special qualities which entitle it to more confidence than we can bestow upon any other agent with which we are acquainted. The vapor, when brought in contact with the odoriferous emanations of gangrene, at once destroy their odor, and probably their specific gangrenous character. When thoroughly applied in sufficient quantity to a gangrenous sore, it at once obliterates all gangrenous odor, and no gangrene appears afterwards, unless the wound receives a new infection from gangrenous matter, allowed to come in contact with it. Its steady vaporization permits it to be applied to the bottom of sinuses and sulci, which cannot be safely laid open with the knife,and which are very common with the patients of this Hospital, and its prompt, almost instantaneous action precludes the necessity of a prolonged use of the remedy, while sufficient experience in its use shows that it may be applied directly to the covering of a nerve, or the periosteum, without destroying their vital integrity.

*Contagiousness.*—Hospital Gangrene is produced by infection upon an exposed surface. Several cases have come to us with the disease following the application of a blister, as well as in bed-sores. One, where the arm was broken, badly-adjusted splints produced abrasion, and gangrene followed. One man acting as nurse to his Captain, who had gangrene, had a little spot of inflammation on his ankle, caused, possibly, by musquito bites, which he had rubbed the skin from, he also had little scratches on his fingers. Gangrene set in at all these points, and he may yet lose his life as the result. In one case at Officers' Hospital, an officer with gangrene occupied a room alone. The carpenters wished to put a water pipe in it, and he was removed into a room where there were three other officers with wounds not then gangrenous. All four had their wounds exposed and dressed, and the gangrenous odor pervaded the apartment. Although the officer was returned in an hour, the next day gangrene appeared in the wounds of all three who had been exposed to the infection. Instances have often occurred in this Hospital where wounds that have been free from gangrene for several days, became again diseased, doubtless from renewed infection, received from others laboring under the disease.

To guard against infection we have made free use of Chlorinated Soda, Chloride of Lime, a solution of the Per-manganate of Potassa, Chlorinum, and Bromine, as well as scrupulous cleanliness and free ventilation. Each patient is supplied with his own drinking cup, and other utensils, and his own sponges; if he has two wounds, and only one is gangrenous, each wound has its sponge, and they are kept as clean as possible. If the nurses, dressers, or surgeons, have wounds or abrasions about their hands, Bromine is applied, and then the hands exposed to the virus without fear. In cleansing a very bad gangrenous sore, the scissors were found to be dull, and while sharpening them I cut out the ball of my left forefinger.

I applied Bromine and the wound healed kindly and readily. Nearly every day I have had cuts, pricks, scratches, or other abrasions on my hands, which are fully exposed in dressing the wounds, and in the dead house, and with no unpleasant consequences, I have always applied Bromine to the denuded surfaces before exposure, and am thoroughly convinced that the virus of the dead house, as well as other animal poisons, is entirely destroyed by the action of Bromine.

*General Treatment.*—The constitution seems to require much more than the usual amount of nutriment in this disease, and it is only after considerable poison has been absorbed, and the heart and lungs have become affected, that digestion and assimilation are deranged. It is true that the *temperature* of the entire body is almost invariably below the normal standard. During the month of August the thermometer ranged in the wards of this Hospital, at 2 o'clock, P. M., at from 98° to 103°, and yet the average temperature of the patients at the same hour, as indicated by the thermometer in their axillar, was but 89°. The average daily temperature of Sergeant Wilkie, who died from pyæmia, after secondary hæmorrhage, was only 74° and that of Private T. D. Riggs, who had gangrene of the right calf, extending down the tendon to the heel bone, was 84°. The pulse of the former averaged 114 beats, and that of the latter 88 beats in a minute This low state of temperature led us to a very free use of alcohol, in the form of ale, porter, whisky, and brandy, usually given once in two hours, and as much nutriment, beef, eggs, etc., as the patient would take. A very marked desire for acids prevailed, which was gratified by giving pickles, cucumbers, citric acid drinks, and acid fruits. After the disease had progressed to the serious invasion of the general system, (and most of our patients were *old* cases brought from other hospitals to die,) a very persistent diarrhœa was common, and that was about the only condition that demanded active medication. We carefully avoided the use of cathartics and opium, and as a soother to nervous and febrile excitement, in place of the opiates, used Hoffman's

Anodyne. In some cases distinctly complicated with miasm, (and most of the patients came from the vicinity of Vicksburg where they had been exposed for months,) we gave the Sulphate of Quinia. One or two doses of ten grains each was, usually sufficient.

*Conditions Favoring the Appearance of Gangrene.*—While no certain *cause*, except contagion, for the disease has been determined, it is evident that a depression of the general vital forces have been favorable to its accession. Fourteen (14) out of forty-six (46) of our patients were secession prisoners, and I think that all of the seven at Officers' Hospital, were also prisoners, a much larger number than a fair proportion, proving that exposure, privation, and consequent depression of the vital forces, is a condition favoring the appearance of the disease.

*Post Mortem Appearances.*—The tables compiled from the Hospital Records, and the reports of individual cases, furnished by Cadets Gunn and Lloyd, together with the learned and able report on the Pathology of the disease, by Surgeon Weeks, who has had unusual opportunities for the study of gangrene at Louisville, Vicksburg, and at this place, preclude the necessity of any extended remarks on this branch of the subject. We have made examinations in every case, with one or two exceptions, of those who have died here, and have observed a very uniform set of conditions. In all we have found thrombi in the heart, some of which have been very large. In one we found a thrombus in the thoracic duct, and I regret that we did not examine that passage in the preceding cases. In one case of long standing, the heart thrombus was softening and the walls of the heart were tainted with gangrene. In another case, where the disease was of still longer duration, the heart thrombus was considerably broken down and liquified, and the walls of the heart very much softened, flabby and decidedly gangrenous.

The lungs in each case were spotted with dark specks, and on being cut into, it was observed that the smaller arteries were often completely filled with small thrombi and dark matter, evidently the debris which had been washed away from a thrombus in the heart, and become lodged in the smaller vessels. The disease was said to have been unusually malignant and rapid here. In no cases have I perceived any collections of pus, or softening of matter, said to have been observed in the lungs of those who have died of gangrene elsewhere.

*Curability of Gangrene.*—This disease, like Syphilis, in its earlier stage, seems to be entirely local, and completely curable, when the parts affected are not vital organs, which has never been primarily in any case that has come under my observation, and when all the parts affected can be reached by direct application of the remedy. But the *secondary* affections, as the thrombi in the heart, the pulmonary complications, and the change in the fluids, which are sometimes called *Pyœmia*, are conditions which do not seem controllable by remedies. We have often had cases recover after the heart, the lungs and the blood had become seriously implicated. But gangrene can be considered certainly curable only in cases where the system has not become seriously affected by disease. In all simple cases gangrene seems as curable as a burn, scald or any other sinus wound.

*General Treatment.*—No remedy has yet been presented to us, that has apparently any marked or positive curative power over the constitutional and organic derangements produced by the absorption of the poison of gangrene. We saturate the air of the wards of the Hospital with Chlorine, and we have given Bromine internally for days and weeks, and while we do not know for a certainty that this course has proved beneficial to our patients, yet we should hesitate to deprive them of even the possible benefits arising therefrom. Pure air, full nutrition, alcoholic preparations, repeated as often as once in two hours, hope, cheerfulness, and immediate removal of all causes of pain, distress, uneasiness, and fretfulness, (for gangrenous patients are very childish,) and perfect cleanliness of the person and the bed, seem preferable to any form of perturbative medication.

